# Risk factors for Korean women to develop an isthmocele after a cesarean section

**DOI:** 10.1186/s12884-018-1821-2

**Published:** 2018-05-15

**Authors:** IY Park, MR Kim, HN Lee, Y Gen, MJ Kim

**Affiliations:** 1 Department of Obstetrics and Gynecology, College of Medicine, Seoul St. Mary’s Hospital, The Catholic University of Korea, Seoul, Republic of Korea; 2 Department of Obstetrics and Gynecology, College of Medicine, Bucheon St. Mary’s Hospital, The Catholic University of Korea, 327 Sosa-ro, Bucheon, Gyeonggi-do 14647 Republic of Korea

**Keywords:** Cesarean section, Isthmocele, Transvaginal ultrasound, Residual myometrial thickness, Uterine flexion

## Abstract

**Background:**

The increase in number of cesarean section (CS) operations has resulted in an increase in cases of isthmocele development. The objective of this study is to determine the risk factors for isthmocele development after CS.

**Methods:**

Isthmocele measurements were taken for 404 women with a history of at least one low transverse CS. The following potential risk factors were investigated: patient’s age at CS, cause of CS, weeks of gestation at CS, premature rupture of membrane (PROM), phase of labor, type suture (single/double layer), operation time, uterine flexion (anteversion/retroversion), and blood transfusion during operation. A transvaginal ultrasound was carried out to examine the isthmocele in the uterus after CS, including the shape of the isthmocele, residual myometrial thickness, depth and width of isthmocele, cervical thickness, location of the isthmocele, and clinical characteristics.

**Results:**

In our study population, the isthmocele had a prevalence of 73.8%. Most isthmocele had a triangular (65.4%) or semicircular shape (10.4%). The presence of an isthmocele was significantly associated with repeat CS, premature rupture of membrane (PROM), short operation time, and extent of cervix dilatation at CS. The risk of isthmocele was low in women who had placenta previa totalis (PPT), twin, a long operation time, or a transfusion during the operation.

**Conclusions:**

In our study, isthmocele development was significantly associated with repeat CS, PROM, a short operation time, and the extent of cervix dilatation at CS. Therefore, PROM prevention and a more careful uterine closure are needed to reduce the risk of developing an isthmocele after CS.

## Background

The number of cesarean section (CS) per 1000 live births in Korea was 380.3 in 2014, which was 1.4 times higher than the average number of CS (264.7 per per 1000 live births) in the Organization for Economic Cooperation and Development (OECD) member countries. It was lower only when compared to that in Turkey (511.3 cases per 1000 live births) [1].

A cesarean-induced isthmocele is a reservoir-like pouch defect on the anterior wall of the uterine isthmus located at the site of a previous cesarean delivery scar [2]. There is no consensus regarding the definition of an isthmocele or a standardized approach for its assessment. The prevalence of an isthmocele in a random population with a history of CS differs between 24 and 70% for transvaginal ultrasound (TVUS) [3]. Although an isthmocele is usually asymptomatic, symptoms related to this condition have been described, and it is a relatively new entity that needs further evaluation.

As the number of CS increases, the number of isthomocele cases has increased, and its associated complications are an important concern. Obstetric complications, such as scar tissue dehiscence, scar pregnancy, and abnormally adherent placenta are associated with this defect. Gynecologic complications due to isthmocele have only recently been identified and described, including abnormal uterine bleeding (postmenstrual spotting), dysmenorrhea, chronic pelvic pain, infertility, adenomyosis, endometriosis, and abscess formation [4]. Three methods exist to conduct a surgical excision of the isthmocele, including hysteroscopic resection, laparoscopic resection and repair with or without robotic assistance, or repair of the isthmocele through vaginal approach [5].

An isthmocele is the result of incomplete healing of the isthmic myometrium after a low transverse uterine incision for CS. However, risk factors to develop the isthmocele after CS are currently unknown. Therefore, this study intends to determine the risk factors for isthmocele in the caesarean scars of Korean women who have undergone caesarean section at a single university hospital.

## Methods

A case control study was carried out with data collected from January 1, 2009 to November 30, 2016 for women with a history of cesarean section (CS) who were screened with transvaginal ultrasound (TVUS) for various gynecological indications. The exclusion criteria included vertical or inverted “T” uterine incision, congenital uterine malformations, fetal death, sepsis, and insertion of intrauterine device. This study was approved by the Ethics Committee of the Catholic University of Korea (approval number: HC17RESI0044). A sonographic assessment of the isthmocele was performed by a single experienced doctor (MJK) using an Accuvix V20 Prestige ultrasound machine (Samsung Medison Co Ltd., Seoul, Korea) equipped with a 4–9 MHz transvaginal probe. The isthmocele was diagnosed when a hypoechogenic area (a filling defect) within the myometrium of the lower uterine segment was present at the site of a previous cesarean incision.

The CS operations had been performed by three doctors with more than 5 years of experience with the operation using a unified single or double layer closure technique with a continuous absorbable suture.

The patients’ clinical information regarding the factors related to isthmocele were collected from medical records, including gestational and maternal age at CS, height, change of weight, BMI, comorbidity, obstetrical history, operation time, repair of uterine incision, duration of active labor, cervical dilatation at CS, type of uterine flexion, and blood transfusion.

The operation time was defined from the skin incision to baby out and from baby out to skin closure according to the anesthetic records. A repair of the uterine incision was performed as a single layer by two operators and a double layer by one operator.

The duration of active labor was classified as follows: without active labor; < 5 h; 5–9 h; and > 10 h. The extent of cervical dilatation was categorized as the following: cervical os was closed; ≤ 4 cm; 5–7 cm; and ≥ 8 cm.

**Fig. 1:**
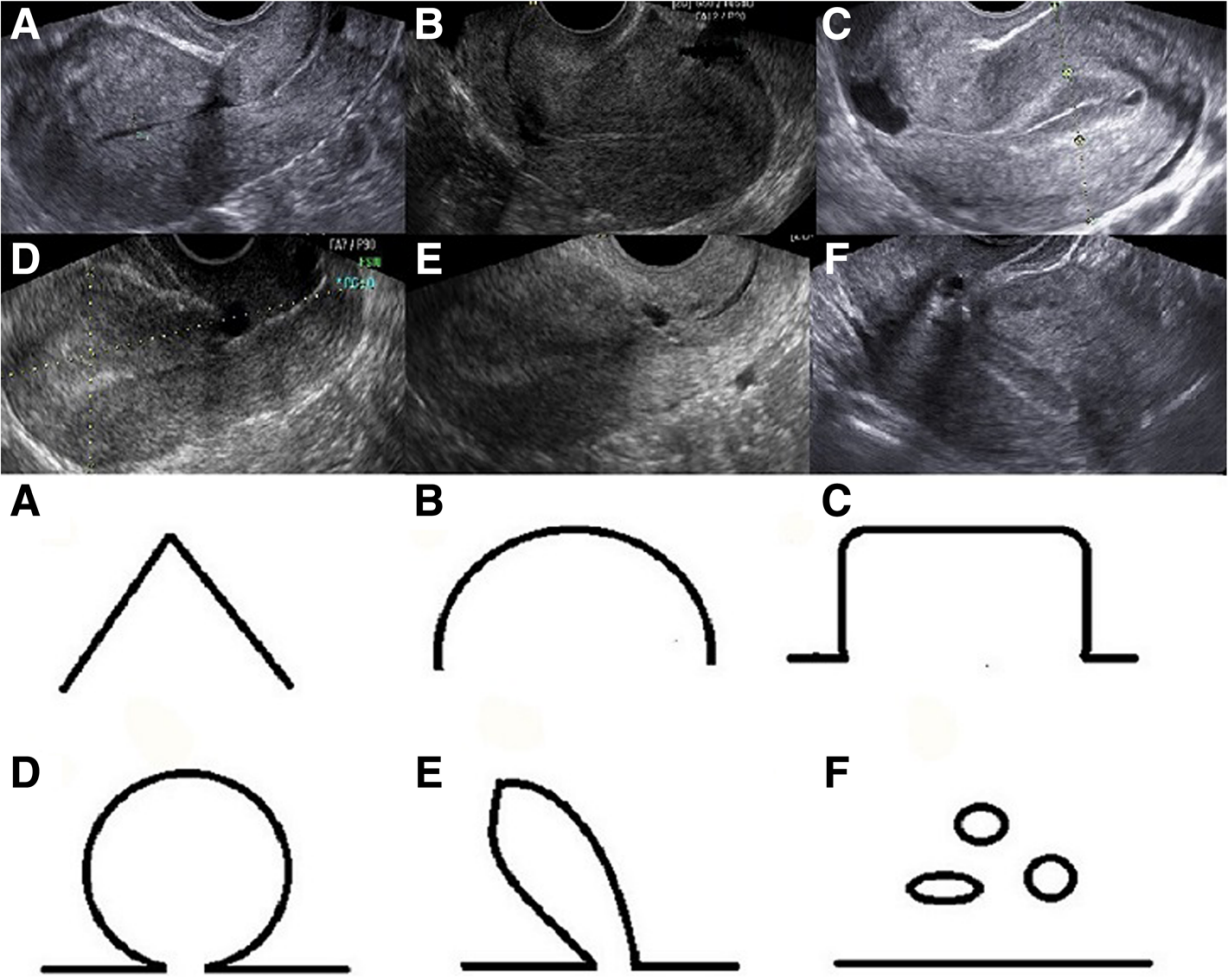
Transvaginal ultrasonography and schematic diagram demonstrating classification used to assess isthmocele shape as **a** triangle, **b** semicircle, **c** rectangle, **d** circle, **e** droplet and **f** inclusion cysts

In this study, the type of isthmocele was categorized into triangle (Fig. 1a), semicircle (Fig. 1b), rectangle (Fig. 1c), circle (Fig. 1d), droplet (Fig. 1e) and inclusion cysts (Fig. 1f), and these types had been used in a previously published study [3].

**Fig. 2:**
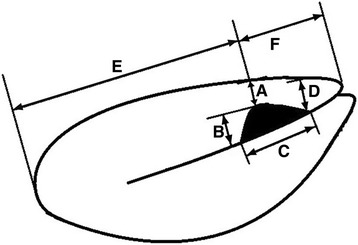
Schematic diagram demonstrating measurement of isthmocele in the longitudinal plane. **a** Residual myometrial thickness; **b** Depth of isthmocele; **c** Width of isthmocele; **d** Cervical thickness; **e** Distance from uterine fundus to isthmocele; **f** Distance from isthmocele to cervix

The isthmocele in the uterus was measured and saved in the longitudinal plane. The residual myometrial thickness (A), depth of the isthmocele (B), width of the isthmocele (C), cervical thickness (D), distance from the uterine fundus to the isthmocele (E), and distance from the isthmocele to the cervix (F) were measured in the sagittal plane (Fig. 2). The residual myometrial thickness (A) was defined as the shortest visible distance in a sagittal plane between the uterine serosal surface and the delineation of the endometrium at the level of the CS scar. TVUS was performed, and the following details of the uterus were recorded: position, length, width, endometrial thickness, and presence of intrauterine fluid.

### Statistical analysis

The data are presented as means ± standard deviation or numbers including percentages based on variables characteristics. An independent t-test was performed for continuous variables while a Chi-square test or Fisher’s exact test was performed for categorical variables. A multivariable analysis of the relationship between the risk of development of an isthmocele and the baseline characteristics was performed using a backward elimination method. Variables displaying *p* < 0.10 based on a univariable analysis were considered as candidates for inclusion in the multivariable logistic analysis. All statistical analyses were performed using SAS software, version 9.4 (SAS Institute Inc., Cary, NC, USA). A two-sided *P* value of less than 0.05 was considered to be statistically significant.

## Results

The study group included 404 women with a mean age of 33.04 ± 1.63 years. The gestational age at CS varied from 28 to 41 weeks with a median value of 37 gestational weeks. A total of 298 (73.8%) women who underwent CS had an isthmocele (the isthmocele group), and 106 (26.2%) women had no isthmocele (without isthmocele group) with intact caesarean scars, including 231 (57.2%) who had underwent CS once, 138 (34.2%) who had underwent CS twice, 28 (6.9%) who underwent CS three times, and 7 (1.7%) who underwent CS four times.

Age, height, weight, BMI, ultrasonographic follow up period, and comorbidity were not significantly different between the two groups (isthmocele group and without isthmocele group). There were no significant differences in the number of CS, parity, abortion, or preterm delivery history between the two groups either. The most frequent gynecological symptoms for visiting the hospital after cesarean section were vaginal spotting and pelvic pain (Table 1).

**Table 1:** Baseline characteristics and the risk factors for presence of an isthmocele

	Without Isthmocele	With Isthmocele	*P* value
Number	106	298	
Age (years)	32.64 ± 4.80	33.19 ± 4.19	0.269
Height (cm)	160.16 ± 4.60	160.24 ± 5.62	0.881
Weight (before pregnancy, kg)	56.74 ± 10.40	58.36 ± 11.66	0.207
Weight (during pregnancy, kg)	70.14 ± 11.18	70.61 ± 11.75	0.720
BMI (before pregnancy, kg/m^2^)	22.12 ± 3.96	22.71 ± 4.23	0.215
BMI (during pregnancy, kg/m^2^)	27.36 ± 4.33	27.47 ± 4.14	0.816
Number of CS (n)	1.43 ± 0.70	1.57 ± 0.70	0.094
Comorbidity (n, %)			
DM	9 (8.5)	30 (10.1)	0.637
Hypertension	14 (13.2)	44 (14.8)	0.695
Parity (n)	1.56 ± 0.74	1.69 ± 0.77	0.127
Preterm experience (n, %)	32 (30.2)	94 (31.5)	0.796
Reason for visiting (n, %)			
Routine examination	65 (61.3)	185 (62.1)	0.890
Vaginal spotting	13 (12.3)	48 (16.1)	0.343
Abnormal uterine bleeding	11 (10.4)	29 (9.7)	0.848
Dysmenorrhea	2 (1.9)	3 (1.0)	0.610
Pelvic pain	14 (13.2)	32 (10.7)	0.492
Sterility	3 (2.8)	1 (0.3)	0.057
Irregular menstruation	3 (2.8)	13 (4.4)	0.772
Follow up period of ultrasound (month)	17.72 ± 25.07	16.38 ± 24.53	0.631
Reason for CS (n, %)			
Repeat CS	34 (32.1)	135 (45.3)	0.018^*^
Fetal distress	14 (13.2)	41 (13.8)	0.887
PPT	15 (14.2)	21 (7.1)	0.028^*^
Preeclampsia	11 (10.4)	23 (7.7)	0.397
Breech	15 (14.2)	25 (8.4)	0.088
Twin	13 (12.3)	10 (3.4)	0.001^*^
Arrest of descent	5 (4.7)	19 (6.4)	0.535
Arrest of dilatation	1 (0.9)	5 (1.7)	1.000
PROM (n, %)	18 (17.0)	83 (28.0)	0.025^*^
Type of CS (n, %)			
Elective CS	59 (55.7)	137 (46.0)	0.087
Emergency CS	47 (44.3)	161 (54.0)	
Gestational weeks	36.72 ± 2.58	36.87 ± 2.74	0.626
Birth weight (g)	2861.9 ± 691.4	2895.2 ± 720.7	0.680
Operation time (total, min)	56.69 ± 18.27	51.88 ± 15.75	0.010^*^
From skin incision to baby out	7.91 ± 5.14	6.78 ± 4.43	0.032^*^
From baby out to skin closure	48.78 ± 15.42	45.10 ± 13.66	0.022^*^
Repair of uterine incision (n, %)			0.579
Single layer	68 (64.2)	200 (67.1)	
Double layer	38 (35.9)	98 (32.9)	
Induction of labor (n, %)	15 (14.2)	55 (18.5)	0.315
Oxytocin augmentation during labor (n, %)	12 (11.3)	56 (18.8)	0.077
Duration of active labor (n, %)			0.218
not in active labor	75 (70.8)	177 (59.6)	
< 5 h	15 (14.2)	62 (20.9)	
5–9	4 (3.8)	18 (6.1)	
≥10 h	12(11.3)	40(13.5)	
With uterine contractions (n, %)	38 (35.9)	137 (46.0)	0.071
Cervical dilatation at CS (n, %)			0.057
closed	72 (67.9)	163 (54.7)	
≤ 4 cm	25 (23.6)	95 (31.9)	
5–7 cm	2 (1.9)	20 (6.7)	
≥ 8 cm	7 (6.6)	20 (6.7)	
Interval of previous CS (years)	1.64 ± 3.14	1.81 ± 2.56	0.610
Uterine position at ultrasound (n, %)			0.205
anteversion	31 (29.3)	114 (38.3)	
retroversion	7 (6.6)	13 (4.4)	

Independent t test was performed for continuous variables, Chi-square test or Fisher’s exact test was performed for categorical variables

*BMI* body mass index, *CS* cesarean section, *PPT* placenta previa totalis, *PROM* premature rupture of membrane

^*^P < 0.05 was considered statistically significant

The shape of isthmocele was categorized into the following 6 groups: triangle, 195 (65.4%); semicircular, 31 (10.4%); rectangle, 25 (8.4%); circle, 22 (7.4%); droplet, 13 (4.4%), inclusion cyst 12(4.0%).

The residual myometrial thickness in the group without isthmocele was 3.08 ± 2.67 mm, which was smaller than that in the group with isthmocele (4.87 ± 3.38 mm) without statistical significance (*P* = 0.099). There was no significant difference in the uterine size, endometrial thickness, or isthmocele location between the two groups. Intrauterine fluid was observed in 47% of patients with an isthmocele. The ratio of E/F (E: distance from uterine fundus to isthmocele; F: distance from isthmocele to cervix) was not significantly different according to the extent of the cervical dilatation or the duration of active labor. Therefore, the location of a cervical incision was not a significant factor to develop the isthmocele (Table 2).

**Table 2:** Characteristics of isthmocele shape

	Without Isthmocele	With Isthmocele	*P* value
Endometrial thickness (mm)	5.48 ± 3.26	5.90 ± 3.00	0.228
Uterine length (mm)	77.24 ± 13.16	77.91 ± 11.15	0.641
Uterine width (mm)	43.29 ± 8.29	43.33 ± 6.83	0.967
Isthmocele size (mm)			
A^a^	3.08 ± 2.67	4.87 ± 3.38	0.099
B^b^	2.72 ± 2.74	4.18 ± 2.41	0.093
C^c^	1.62 ± 1.70	6.24 ± 3.48	<.001^*^
D^d^	9.31 ± 2.98	9.95 ± 3.13	0.191
E^e^	50.06 ± 9.19	51.65 ± 9.68	0.145
F^f^	27.03 ± 6.76	26.83 ± 7.42	0.814
Ratio (E/F)	1.97 ± 0.63	2.11 ± 0.85	0.076

^a^Residual myometrial thickness

^b^Depth of isthmocele

^c^Width of isthmocele

^d^Cervical thickness

^e^Distance from uterine fundus to isthmocele

^f^Distance from isthmocele to cervix

^*^P < 0.05 was considered statistically significant

Table 3 presents the results of a logistic regression analyzing the associations between isthmocele and the risk factors. The risk of the isthmocele increased with repeat CS (odds ratio/OR: 2.30, 95% CI: 1.26–4.23; *P* = 0.007), PROM (OR: 1.90, 95% CI: 1.08–3.34; *P* = 0.027), cervical dilatation (1–4 cm and 5–7 cm) at CS (OR: 3.04, 95% CI: 1.57–5.92; *P* = 0.001; OR: 13.11; 95% CI: 1.77–97.27; *P* = 0.012, respectively).

**Table 3:** Association between isthmocele and demographic background variables

	Univariable		Multivariable	
	Odds ratio (95% CI)	*P* value	OR (95% CI)	*P* value
Age (years)	1.03 (0.98–1.08)	0.268		
Number of CS	1.34 (0.95–1.88)	0.095		
BMI (before pregnancy)	1.04 (0.98–1.10)	0.216		
BMI (during pregnancy)	1.01 (0.95–1.06)	0.815		
Parity	1.27 (0.93–1.72)	0.128		
Preterm experience	1.07 (0.66–1.72)	0.796		
Reason for CS				
Repeat CS	1.75 (1.10–2.80)	0.019^*^	2.30 (1.26–4.23)	0.007^*^
Fetal distress	1.05 (0.55–2.01)	0.888		
PPT	0.46 (0.23–0.93)	0.031^*^		
Preeclampsia	0.72 (0.34–1.54)	0.399		
Breech	0.56 (0.28–1.10)	0.091		
Twin	0.25 (0.11–0.59)	0.001^*^	0.25 (0.11–0.59)	0.001^*^
Arrest of descent	1.38 (0.50–3.78)	0.537		
Arrest of dilatation	1.79 (0.21–15.52)	0.596		
PROM	1.90 (1.08–3.34)	0.027^*^		
Type of CS				
Elective CS	Reference		Reference	
Emergency CS	1.48 (0.95–2.30)	0.087		
Pregnancy (weeks)	1.02 (0.94–1.11)	0.625		
Birth weight	1.00 (1.00–1.00)	0.679		
Operation time (total, min)	0.98 (0.97–1.00)	0.011^*^	0.97 (0.96–0.99)	0.002^*^
From skin incision to baby out	0.95 (0.91–1.00)	0.037		
From baby out to skin closure	0.98 (0.97–1.00)	0.024		
Repair of uterine incision				
Single layer	Reference		Reference	
Double layer	0.88 (0.55–1.40)	0.579		
Induction of labor	1.37 (0.74–2.55)	0.316		
Oxytocin augmentation during labor	1.81 (0.93–3.53)	0.081		
Duration of active labor		0.224		
not in active labor	Reference		Reference	
< 5 h	1.75 (0.94–3.27)	0.079		
5–9 h	1.91 (0.62–5.82)	0.257		
≥ 10 h	1.41 (0.70–2.84)	0.333		
With uterine contractions	1.52 (0.96–2.41)	0.072		
Cervical dilatation at CS		0.072		0.002^*^
closed	Reference		Reference	
≤ 4 cm	1.68 (1.00–2.83)	0.051	3.04 (1.57–5.92)	0.001^*^
5–7 cm	4.41 (1.01–19.37)	0.049	13.11 (1.77–97.27)	0.012^*^
≥ 8 cm	1.26 (0.51–3.12)	0.614	2.04 (0.71–5.91)	0.188
Ratio (E/F)	1.27 (0.94–1.72)	0.124		
Blood transfusion	0.33 (0.14–0.79)	0.013^*^		

Multivariable logistic regression model was performed by backward elimination methods. Because of missing data, some isthmocele variables were not calculated

^*^P < 0.05 was considered statistically significant

The risk of an isthmocele was low in those with placenta previa totalis (PPT) (OR: 0.46, 95% CI: 0.23–0.93; *P* = 0.031), twin (OR: 0.25, 95% CI: 0.11–0.59; *P* = 0.001), long operation time (OR: 0.97, 95% CI: 0.96–0.99; *P* = 0.002), and transfusion (OR: 0.33, 95% CI: 0.14–0.79; *P* = 0.013).

After CS, there was no correlation between the changes in the uterine direction (anterflexion/retroflexion) and the occurrence of an isthmocele (Table 4).

**Table 4:** Relationship between the presence of isthmocele and change in uterine flexion from early pregnancy to post CS state

	Without Isthmocele (*n* = 38)	With Isthmocele (*n* = 127)	*P* value
Flexion changed	8 (21.1%)	37 (27.1%)	0.326
from A to P	8 (21.1%)	33 (26.0%)	0.537
from P to A	0 (0.0%)	4 (3.2%)	0.575
Flexion did not change	30 (79.0%)	90 (70.9%)	0.326
from A to A	23 (60.5%)	81 (63.8%)	0.716
from P to P	7 (18.4%)	9 (7.1%)	0.057

Comparisons of data were performed by Chi-squared test and significance was considered at *P* < 0.05

*A* Anteflexion

*P* Retroflexion

## Discussion

An isthmocele is a common incidental finding on TVUS that is usually asymptomatic (the prevalence of a symptomatic isthmocele remains unknown). Morris first described cesarean-delivery scar defects, including utero peritoneal fistula, niche, and isthmocele in 1995 [5]. Ofili-Yebovi et al. [6] described this finding as a “detectable myometrial thinning at the site of the Cesarean section scar”. This defect is also referred to as a “cesarean scar defect”, “uterine scar defect”, “uterine diverticulum”, “niche”, “isthmocele”, “pouch”, or “sacculation” [7].

When diagnosing an isthmocele, an important consideration is whether a radiologic identification correlates with clinical symptoms. Patients with an isthmocele often present spotting after menstruation due to the accumulation of blood in the defect of the uterus. The best time to perform TVUS is in the early follicular phase when the accumulation of blood within the defect enables its detection [8]. Some women with isthmocele also experience pelvic pain, vaginal discharge, dysmenorrhea, and dyspareunia.

The most ischemic process change and slowest absorbable suture might be the worst combination, and they are more likely to produce isthmocele. The occurrence of a defective uterine scar after cesarean section depends on multiple factors, including the degree of cervical dilatation and possibly the contractile effort of the uterine musculature, resulting in thinning at the uterine incision site [2].

Between 2009 and 2014, the number of CS per 1000 births per 1000 OECD populations increased by 18.1. In Korea, there was an increase of 16.5 between 2009 and 2013 (OECD Health Statistics 2017, Ministry of Health and Welfare). As the number of cesarean deliveries increases, efforts are needed to reduce isthmocele-related complications after cesarean section.

The diagnosis of isthmocele is based on TVUS or hysteroscopy. The use of TVUS to diagnose a cesarean scar was reported in 1990, with the following four key sonographic findings: a wedge defect, inward protruding of the scar, outward protruding and hematoma, or retraction of the scar [9]. Others have described a cesarean scar on TVUS as a triangular anechoic area with the apex pointing anterior or a filling defect on the anterior isthmus [4, 8]. In our study, the type of isthmocele in TVUS was divided into triangle (65.4%), semicircle, rectangle, circle, and droplet and inclusion cysts.

The prevalence of isthmocele ranges from 24 to 70% when assessed by TVUS and from 56 to 84% when assessed by sonohysterography [10]. The frequency of the isthmocele on an ultrasonography was 73.8% in a random population of Korean women with a history of CS in the present study.

The occurrence of isthmocele was not associated with maternal age, change of BMI, parity, or preterm experience in our study. However, some studies reported that isthmocele development is associated with age, BMI, preeclampsia, post-operative anemia and WBC count [11, 12].

A history of multiple cesarean deliveries is associated with wider and larger isthmocele as the main risk factor for isthmocele development [4, 6, 11, 12]. However, our study showed different results. Although the risk of an isthmocele increased when repeated cesarean section was performed (OR: 2.30, 95% CI: 1.26–4.23, *P* = 0.007), the number of CS did not increase the risk of isthmocele (OR: 1.34, 95% CI: 0.95–1.88, *P* = 0.095). An isthmocele was more frequent in the group with repeated cesarean section than in that with its first CS operation, and it was not related to the increase in the number of cesarean section. Therefore, reducing CS incidence is an important factor to reduce the risk of isthmocele.

PROM is a major risk factor of infection and is known to weaken healing in uterus closure [12, 13]. PROM patients might have an immature lower segment of the uterus, which might be detrimental to uterine suture and wound healing. Hayakawa et al. [13] reported that premature rupture of the membranes increases the risk of isthmocele. Our study also showed that the risk of isthmocele increased by 1.9 (95% CI: 1.08–3.34) in those with PROM. Therefore, appropriate management should be taken to prevent infection and to facilitate proper healing and remodeling of the myometrial incision if PROM is accompanied during delivery.

Emergency CS and the presence of labor are not risk factors for the presence of an isthmocele [13, 14]. Our results also indicated that emergency surgery and coexisting uterine contractions did not increase the risk of isthmocele.

During cesarean section, a uterine suture can be performed using the single- or double-layer method. The relationship between the different types of uterine closure and the prevalence of cesarean scar defects is currently unclear. A prospective cohort study reported that large niches are more frequent in women with one-layer uterine closure (90.9%) when compared to those in a two-layer closure (9.1%) [15]. However, the difference was not statistically significant [15]. In our study, 268 cases (66.3%) were sutured in a single layer and 136 cases (33.7%) were performed in a double layer. The OR for isthmocele development was 0.88 (0.55–1.40) in the double-layer suture, which was not statistically significant.

A cesarean section during active labor with cervical dilatation over 5 cm is associated with a large cesarean scar defect [15]. The association between a low uterine incision and the development of a niche during active labor can be explained by a thin myometrium and impaired healing [10, 15, 16]. The risk of a large niche increases if the station of the presenting part of the fetus at CS is below the pelvic inlet, cervical dilatation is ≥5 cm, or duration of labor was ≥5 h [15]. Incomplete healing of the uterine scars in cases of CSs performed during advanced labor might be a result of the incision through cervical tissue because the cervix becomes incorporated into the lower uterine segment in the physiological process of cervical effacement [17]. In the present study, the risk of isthmocele increased when the uterine cervix was dilated to less than 7 cm. However, when the cervix was dilated to more than 8 cm, it did not increase the risk of isthmocele. The operator might have intentionally performed the incision at the upper side when the uterine cervix was completely dilated. Although those without a scar defect had a significantly higher scar location above the internal cervical os than those with a scar defect [18], the cause of this result is unclear. The effect of the location of the incision in the uterus might be difficult to study, so further research is needed to confirm the hypothesis that the cervical location of the uterine scar impairs wound healing.

For the first time, this study investigated the association between isthmocele risk and the operation time based on the anesthetic record. The operating time was divided into skin incision to baby out and baby out to skin closure. The multivariate logistic analysis showed that the isthmocele was less occurred with a longer operation time (OR: 0.97, 95% CI: 0.96–0.99, *P* = 0.002). Shortening the operation time seems to affect the occurrence of an isthmocele. Although a definite mechanism for this is uncertain, a more careful uterine closure and bleeding control may be important if the procedure is performed over a short period of time.

In our study, repeat CS, PROM, the extent of cervical dilatation at CS, and short operation time were factors associated with the occurrence of isthmocele whereas PPT, twin, and blood transfusion during operation did not increase the risk of isthmocele. Although PPT, cervical lacerations and extensive blood loss (blood transfusion) causing surgical difficulties could affect the healing process of the myometrial incision site, more careful attention (by the operator) during procedures could be performed. Manipulations such as uterine suture, bleeding control, and uterotonics (carbetocin, methergine, oxytocin etc.) decreased the incidence of an isthmocele. However, transfusions only occurred in 21 cases (small sample size), and this is a limitation of our study, so further study is needed.

The association between a retroverted uterus and isthmocele occurrence or extent was evaluated in other studies [4, 6, 11, 12]. A suggested explanation for this association is that the hysterotomy in a retroverted uterus might have greater tension during healing, leading to mechanical traction and impaired perfusion [6]. A change in the uterine flexion after CS could be due to surgery-related factors, such as a low (cervical) location of the uterine incision during CS, incomplete closure of the uterine wall due to single-layer suture, endometrial saving closure technique or the use of locking sutures, and surgical activities that may induce adhesion formation [18]. It is currently unclear whether a retroflexed uterus that causes the scar to heal improperly is a reason or the retroflection is the result of isthmocele itself due to the lack of support of the corpus by the incomplete closure of the uterine wall. Our results revealed no relationship between the flexion of the uterus and the occurrence of isthmocele in preoperative or postoperative changes.

It is not known whether an isthmocele during an ultrasound examination in nonpregnant women developed a greater risk for complications in subsequent pregnancies than intact scars, or whether large defects are related to greater risk compared to small defects.

Treatment of symptomatic women with an isthmocele ranges from hormonal treatment to hysterectomy, and a failure of hormonal treatment for abnormal uterine bleeding in women with isthmocele eventually required surgical treatment. However, there has been no evidence that surgical correction for an isthmocele improves obstetric outcomes [17].

Although the most important thing to reduce the incidence of isthmocele is to reduce the incidence of CS, it is important to know which factors impair proper wound healing in order to prevent the formation of an isthmocele when CS is needed. The results of this study could be used to guide future research to elucidate the etiology of isthmocele development. A standard definition of an isthmocele and an assessment method should be defined in the future to enable meta-analyses and further research on risk factors to prevent isthmocele development.

## Conclusion

In conclusion, the presence of an isthmocele was significantly associated with repeat CS, PROM, short operation time, and extent of cervix dilatation at CS. However, the risk of isthmocele was low in women who had PPT, twin, long operation time, or transfusion during operation. Considering the increasing incidence of cesarean section, PROM prevention, longer operation time, and more careful uterine closure are needed to reduce the risk isthmocele development after CS.

## Abbreviations

CS: Cesarean section
OR: Odds ratio
PPT: Placenta previa totalis
PROM: Premature rupture of membrane
TVUS: Transvaginal ultrasound
